# The role of kinesin family members in hepatobiliary carcinomas: from bench to bedside

**DOI:** 10.1186/s40364-024-00559-z

**Published:** 2024-03-03

**Authors:** Kai Zhao, Xiangyu Li, Yunxiang Feng, Jianming Wang, Wei Yao

**Affiliations:** 1 Department of Biliary and Pancreatic Surgery, Cancer Research Center Affiliated Tongji Hospital, Tongji Medical College, Huazhong University of Science and Technology, 430030 Wuhan, Hubei China; 2 Department of Thoracic Surgery Affiliated Tongji Hospital, Tongji Medical College, Huazhong University of Science and Technology, 430030 Wuhan, Hubei China; 3 Affiliated Tianyou Hospital, Wuhan University of Science & Technology, 430064 Wuhan, China; 4 Department of Oncology Affiliated Tongji Hospital, Tongji Medical College, Huazhong University of Science and Technology, 430030 Wuhan, Hubei China

**Keywords:** Kinesin, Hepatobiliary carcinoma, Signaling transduction, Prognosis assessment, Targeted therapy

## Abstract

As a major component of the digestive system malignancies, tumors originating from the hepatic and biliary ducts seriously endanger public health. The kinesins (KIFs) are molecular motors that enable the microtubule-dependent intracellular trafficking necessary for mitosis and meiosis. Normally, the stability of KIFs is essential to maintain cell proliferation and genetic homeostasis. However, aberrant KIFs activity may destroy this dynamic stability, leading to uncontrolled cell division and tumor initiation. In this work, we have made an integral summarization of the specific roles of KIFs in hepatocellular and biliary duct carcinogenesis, referring to aberrant signal transduction and the potential for prognostic evaluation. Additionally, current clinical applications of KIFs-targeted inhibitors have also been discussed, including their efficacy advantages, relationship with drug sensitivity or resistance, the feasibility of combination chemotherapy or other targeted agents, as well as the corresponding clinical trials. In conclusion, the abnormally activated KIFs participate in the regulation of tumor progression via a diverse range of mechanisms and are closely associated with tumor prognosis. Meanwhile, KIFs-aimed inhibitors also carry out a promising tumor-targeted therapeutic strategy that deserves to be further investigated in hepatobiliary carcinoma (HBC).

**Supplementary Information:**

The online version contains supplementary material available at 10.1186/s40364-024-00559-z.

## Introduction

Hepatobiliary carcinomas include primary hepatocellular carcinoma (HCC), cholangiocarcinoma (CCA) and gallbladder cancer (GBC). Generally, HCC accounts for ~ 90% of liver cancers, having a five-year survival rate of approximately 18% and being the third most frequent cause of cancer-associated death [1, 2]. Indeed, the number of cases of HCC is projected to exceed one million by 2025, implying it remains an intractable health challenge globally [2]. Ranking behind HCC in primary liver cancers, CCA occupies ~ 3% of total gastrointestinal malignancies, and inferior to HCC, despite advancements in cognization, diagnosis, and management of CCA, the overall prognosis remains unsatisfactory with a five-year survival rate of about 7–20% [3]. As for GBC, the poor prognosis represents its most striking feature that less than 5% of patients survive for five years, perhaps due to the atypical clinical manifestations which make early diagnosis difficult [4, 5]. Indicated by these statistics, the exploration and development of effective diagnostic and therapeutic strategies for these three types of HBCs remains urgent needed.

As one essential category of motor proteins, KIFs are responsible for intracellular trafficking in a highly spatially and temporally regulated manner, whose function depends on their conservative microtubule-reliant motion feature and adenosine triphosphatase (ATPase) activity [6, 7]. Under physiological conditions, KIFs-mediated dynamic organelle and chromosome transport are critical for cell division and genetic homeostasis. Dysregulation of KIFs contributes to multiple diseases, including Neurodegenerative diseases associated with defective axonal transport such as amyotrophic lateral sclerosis (ALS) [8, 9], hereditary spastic paraplegia (HSP) [10], and Alzheimer’s disease [11], as well as illnesses caused by dysfunctional transport complex, such as polycystic kidney disease [12], retinitis pigmentosa [13]. Furthermore, not surprisingly, KIFs have also been linked to tumorigenesis [14].

However, the roles of KIFs in HBC have not been clearly defined. The current work gives an overview of the kinesin family members, indicating the relevance to HBC progression and the potential for prognostic evaluation. Meanwhile, KIFs-targeted therapeutics are also reviewed with the aim of illustrating the feasibility for the treatment of hepatobiliary solid tumors.

## Overall landscape of kinesin superfamily

**Fig. 1:**
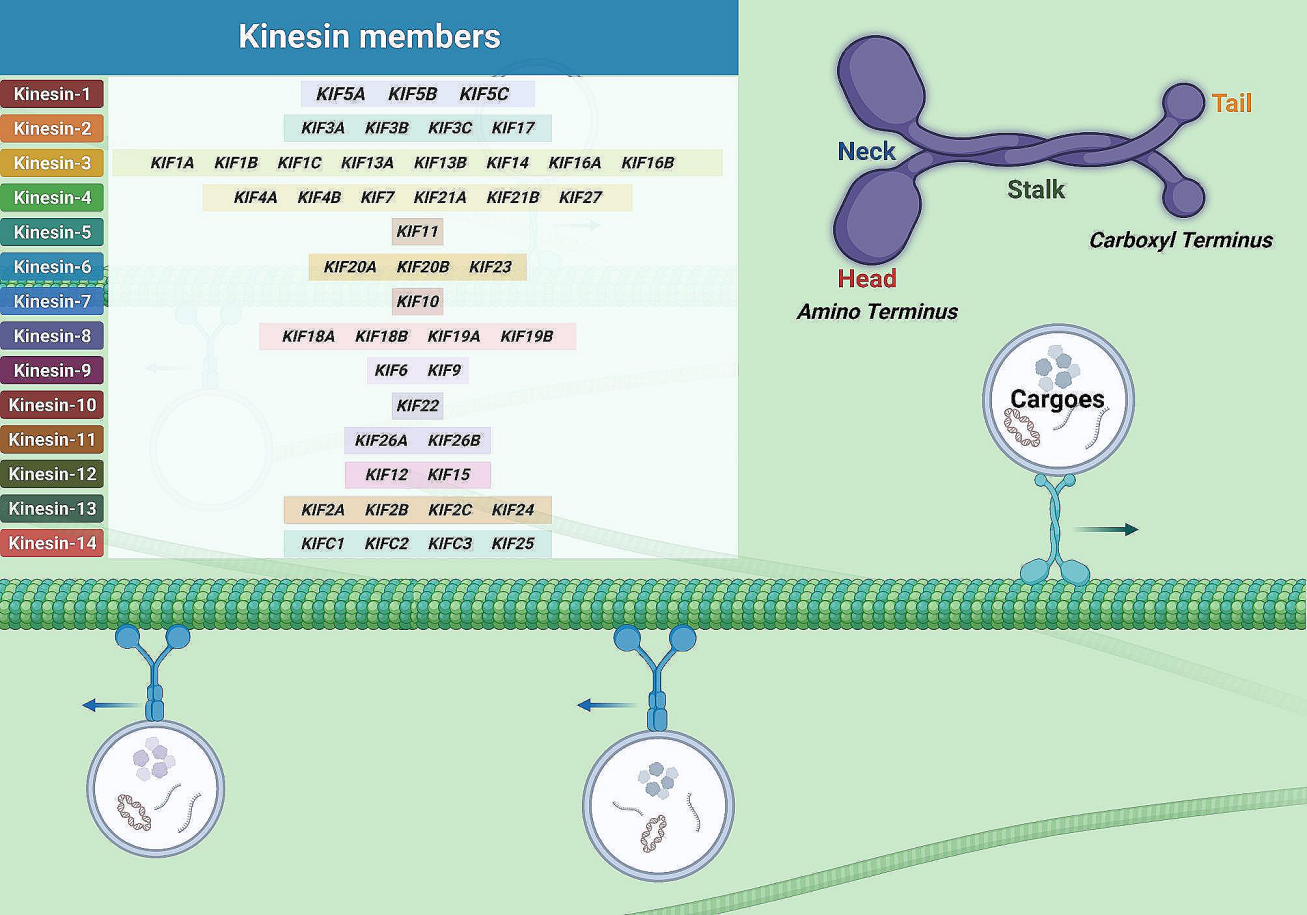
Overall landscape of kinesin superfamily. Genes for 45 different kinesins belonging to 14 subfamilies have been identified in the murine and human genomes. Kinesin structure incorporates four primary domains: head, neck, stalk, and tail. The amino and carboxyl termini are labeled

Microtubule-dependent intracellular transport by KIFs was first described in the 1980s. Quick-freezing and deep-etching (QF-DE) technology allowed the identification of fine cross-linked structures between organelles and microtubules, of which kinesins belonged to an important category. Kinesins possessed both ATPase activity and motion peculiarity, were responsible for driving transportation in a particular direction along microtubules [15–18]. Until now, a total of 45 diverse KIFs classified into 14 different subgroups (from kinesin-1 to 14) have been acknowledged in the murine and human genome [19, 20] (Fig. 1). However, due to the existence of mRNA alternative splicing, each kinesin gene may correspond to 2–3 diverse mRNAs, leading to much more KIFs-translated proteins than original gene counts [21]. Therefore, further exploration remains necessary to determine the detailed number of KIF subtypes and clarify their specific cellular functions.

### The biological function of kinesin superfamily

As the motor proteins with a high degree of conservation, the microtubule-guided movement of KIFs actuates multiple biological activities, including intracellular transport, mitosis, meiosis, as well as signaling transduction [7, 17]. Structurally, all KIFs incorporate a highly conserved head motor domain, and according to its molecular position, the KIFs can be further classified into three major categories: N-KIFs with an amino-terminal motor domain, C-KIFs with a carboxyl-terminal motor domain, and M-KIFs in which the motor domain is located in the middle. Attaching to the head motor domain is a short and flexuous segment, figuratively called the neck domain, followed by a central, long coiled-coil domain and a tail region [22–25]. Functionally, the head motor domain possesses both ATP-combining and microtubule-binding sites, responsible for providing energy for the mechanical movement along microtubules [23, 26]. Meanwhile, the differential location of the motor domain determines transport direction, with N-KIFs actuating plus end-directed transport along microtubules, C-KIFs mediating minus end-directed motility, and M-KIFs being responsible for microtubule depolymerization [7]. Unlike the conserved motor domain, the connected neck domain shows great variability among kinesin families and influences the direction of movement of the protein [27]. As the central region in the whole structure of kinesins, the coiled-coil (stalk) domain mediates inter-subunit cross-interaction of the holoenzyme as well as the formation of the kinesin dimer. And the tail domain is primarily worked to interact with the kinesin-carrying cargoes, such as vesicles, organelles, and protein complexes [23, 28, 29]. Despite the conservative property of the head motor domain among distinct kinesins, the sequence variability of the stalk and tail domains allows flexibility in function which ranges from neurotransmitter transport during synaptic signaling transduction to chromosomal transport during cell proliferation and division [30].

### The regulation mechanism of kinesins in tumor pathogenesis

Considering the important biological functions mediated by KIFs in cell initiation and evolution, it seems unsurprising that aberrant KIFs may contribute to cancer development, indeed, numerous studies have indicated this hypothesis [31–40]. Tracing the reason, KIFs function is central to the integrity of spindle formation, chromosomal segregation, and cytokinesis during normal cell proliferation and genetic homeostasis, while aberrant KIFs may destroy these processes, leading to abnormal spindle assembly, insufficient cytokinesis, chromosomal aneuploidy, and mitotic retardation, precipitating the unequal distribution of hereditary information and homeostatic imbalance, eventually inducing tumor initiation and progression [41–43].

### Implications of tumor management targeting KIFs

In view of the essential status of aberrant KIFs in tumorigenesis, figuring out their specific functions seems quite important and necessary. On the one hand, the abnormalities in KIFs expression may be specific to cancer type, implying that testing of KIFs mRNA or protein may be a tool for early diagnosis and prognostic assessment for individual cancers. On the other hand, given the implicated roles of KIFs in signaling transduction, KIFs-mediated tumor progression may be due to disruption of normal KIFs signaling roles, therefore, contraposing particular KIFs also exposes the possibility of novel anti-tumor targets which may assist with the common problem of resistance to currently available chemotherapy.

Indeed, there already have extensive explorations of KIFs in the context of many human cancers, as well as achievements in KIFs-targeted small-molecule drug development [44]. However, the situation is complicated by the large number of KIF genes and the possibility of further variation generated by mRNA alternative splicing, our current cognization about KIFs appears insignificant. Especially for solid tumors originating from the hepatobiliary system, despite the advancement in surgical treatment during the past decades, patients can benefit significantly from radical section of the lesion, the long-term survival remains disappointing, partly due to the limited availability of adjuvant therapy [45, 46]. HBCs show great genetic heterogeneity and exist in an immunosuppressive microenvironment, limiting the effectiveness of currently-available chemo- and immuno-therapeutics, thereby seeking and exploiting valuable therapeutic targets is a protracted battle for comprehensive cancer treatment, particularly for hepatobiliary malignancies [47, 48].

Here, we carried out this work to make a deep understanding of KIFs. A detailed summary follows of their current status in HBCs from hepatocellular carcinoma to biliary tract cancers, which aims to address KIFs specific functions and the potential for diagnostic and prognostic utility. Additionally, KIFs-targeted small molecule agents are also described with a view to exposing novel therapeutic strategies for HBCs.

## The roles of KIFs in HCC

Aberrant KIFs can impel or restrict the pernicious progression of HCC. In this section, we made a retrospective summary of the reported biological functions of KIFs as well as the associated dysfunctional signaling in the development of HCC following the family order, aiming to furnish the correlated theoretical basis for HCC management via targeting KIFs.

### Kinesin-2 family

**Fig. 2:**
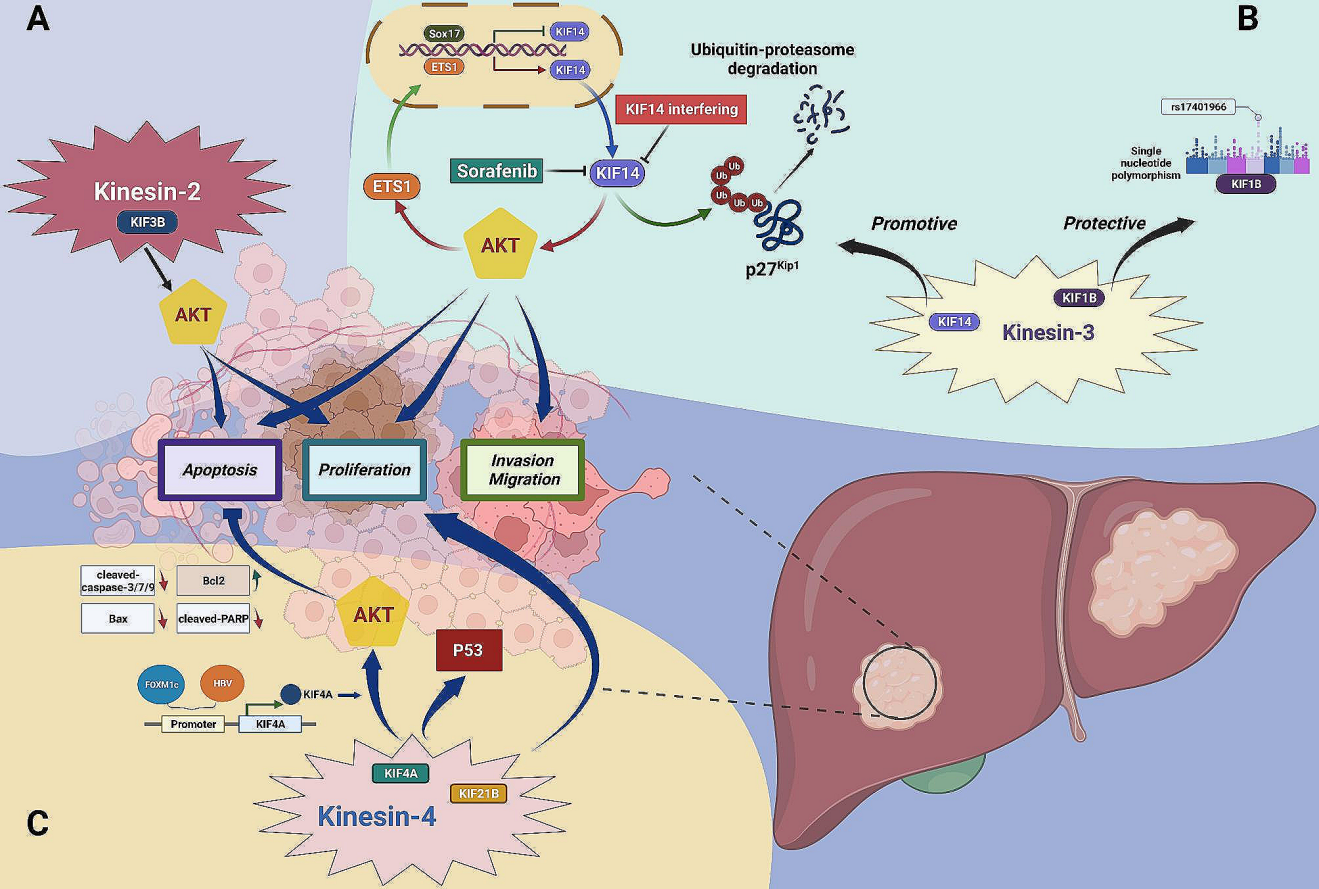
The roles of the kinesin-2, 3, 4 family members in HCC. (**A**) Kinesin-2 family: KIF3B may influence tumor cells proliferation and apoptosis via an effect on Akt signaling pathway. (**B**) Kinesin-3 family: Tumor promotion: a positive feedback loop has been described between KIF14 and ETS1 in Akt signaling transduction. Transcription factor ETS1 up-regulates KIF14 expression, and KIF14 promotes tumor development by stimulating Akt signaling, which subsequently activates ETS1 to further magnify this effect. Both sorafenib treatment, KIF14 interference, and transcriptional suppression by Sox17 can disrupt the cycle by inhibiting KIF14 expression. Moreover, the tumor promotion mediated by KIF14 may also be correlated with post-translational modification (ubiquitination) of p27^Kip1^. Tumor suppression: the single nucleotide polymorphism (SNP) of KIF1B (represented by rs17401966) has been reported to protect against HCC. (**C**) Kinesin-4 family: KIF4A protects tumor cells from apoptosis by stimulating PI3K/Akt signaling, and both FOXM1c and hepatitis B virus (HBV) can positively regulate KIF4A expression at the transcriptional level. And two underlying associations: KIF4A and p53 signaling, KIF21B and tumor cell proliferation and survival, remain to be further investigated

First for the kinesin-2 family shown in Fig. 2A, there was only one study that reported the promotive role of KIF3B in HCC pathogenesis, KIF3B knockdown could drastically restrain proliferation and stimulate apoptosis of cancer cells, and this effect was probably correlated with the Akt signaling given the result that the reduction of p-Akt expression following KIF3B-depletion [49]. However, the specific roles of KIF3A, KIF3C, and KIF17 from the same family as well as their possible carcinogenic approaches are not clear yet in HCC.

### Kinesin-3 family

For the kinesin family with the greatest number of subtypes, their influences on tumors are of multiple phenotypic dimensions. For example, it was observed that the expression of KIF14 was elevated in sorafenib-resistant hepatoma cell lines, HepG2 and Huh7, in which sorafenib treatment had a much lessened inhibitory effect on KIF14 relative to parent cells. Indeed, KIF14-silencing markedly enhanced the sorafenib-sensitivity of tumor cells, which could be further bolstered through the combination of KIF14-intervention and sorafenib treatment, implying that this protein may underlie sorafenib resistance in HCC. Mechanically speaking, dysfunctional Akt signaling has been linked to sorafenib resistance, KIF14-inhibition efficaciously attenuated p-Akt expression thereby suppressing Akt signaling transduction and reversing the acquired resistance to sorafenib. Moreover, as the crucial downstream transcriptional factor of PI3K/Akt pathway, ETS1 was inhibited by sorafenib and closely associated with sorafenib-resistant, the research further demonstrated that there was a feedback regulation loop between KIF14 and ETS1, silencing either of them could significantly reduce the expression of the other, thus suppressing Akt signaling and reducing sorafenib resistance, implying the important role of this positive feedback loop in the sorafenib-resistant induction. Regression to the clinical work, the combination of KIF14 knockdown and sorafenib treatment represents a promising therapeutic strategy for patients with HCC, especially for those sorafenib-tolerated [50]. In addition to sorafenib, restraining the expression of KIF14 by small interfering RNA (siRNA) was also found to promote cisplatin sensitivity, another common cytotoxic regent in HCC chemotherapy [51]. Meanwhile, KIF14 was also involved in the modulation of conventional malignant behaviors like cell division, proliferation, migration, and apoptosis, which might also depend on PI3K/Akt signaling [52], or be related to the post-translational modification of p27^Kip1^ mediated by KIF14-silencing [53]. Furthermore, besides the exogenous KIF14 inhibition, transcription of the KIF14 gene may also be directly suppressed by Sox17, implicating a possible alternative approach for preventing HCC progression via targeting KIF14 [54].

By contrast, unlike the carcinogenic role of KIF14, another family member KIF1B has been reported to exert a protective role in HCC pathogenesis [55]. Relevant research mainly concentrated on the association between the KIF1B single nucleotide polymorphism (SNP) (represented by rs17401966) and susceptibility of HBV-related HCC carcinogenesis [56–58], noting that the polymorphisms of KIF1B (rs17401966) might be tightly connected with a protective effect against HCC, especially in HBV-infected individuals and the Chinese population [59–61]. However, such results have proved controversial [62–65], and large-scale and multi-population cohort research remains desired to further investigate and clarify the situation. Nevertheless, these observations also provide new insights into the influence of heritable variation on HCC pathogenesis (Fig. 2B).

### Kinesin-4 family

Except for the regulatory roles of kinesin-3 family members in Akt signaling, KIF4A (kinesin-4 family member) was also confirmed to protect tumor cells from apoptosis via facilitating PI3K/Akt signaling, reflected by the up-regulation of p-Akt levels, as well as corresponding changes of several apoptosis biomarkers when KIF4A was overexpressed, including decreased Bax, cleaved-caspase-3/7/9, cleaved-PARP, and increased Bcl2, while these promoting effects could be reversed by KIF4A depletion [66]. Meanwhile, KIF4A was also found to be an immediate downstream target of FOXM1c, an isoform of the complicated tumor-associated transcription factor FOXM1, which could directly bind with the promoter of KIF4A and positively regulate its expression, thereby stimulating tumor growth both in vitro and in vivo [67]. Analogously, KIF4A could also be transcriptional activated by hepatitis B virus (HBV) in a dose-dependent manner, and this connection perhaps assisted in explaining the pathogenesis of HBV-related HCC from a novel perspective [68]. Besides the above KIF4A-involved signaling axis revealed by the experimental evidence, bioinformatic analysis has also noted a link between KIF4A and p53 signaling, while deep exploration remains necessary to further discover this potential regulatory association [69].

And KIF21B, another kinesin-4 family member, also mediated HCC carcinogenesis by facilitating tumor cells proliferation and survival. Regrettably, investigations regarding the underlying oncogenic mechanism of KIF21B in HCC are also insufficient that further elucidation remains wanted [70] (Fig. 2C).

### Kinesin-5 family

**Fig. 3:**
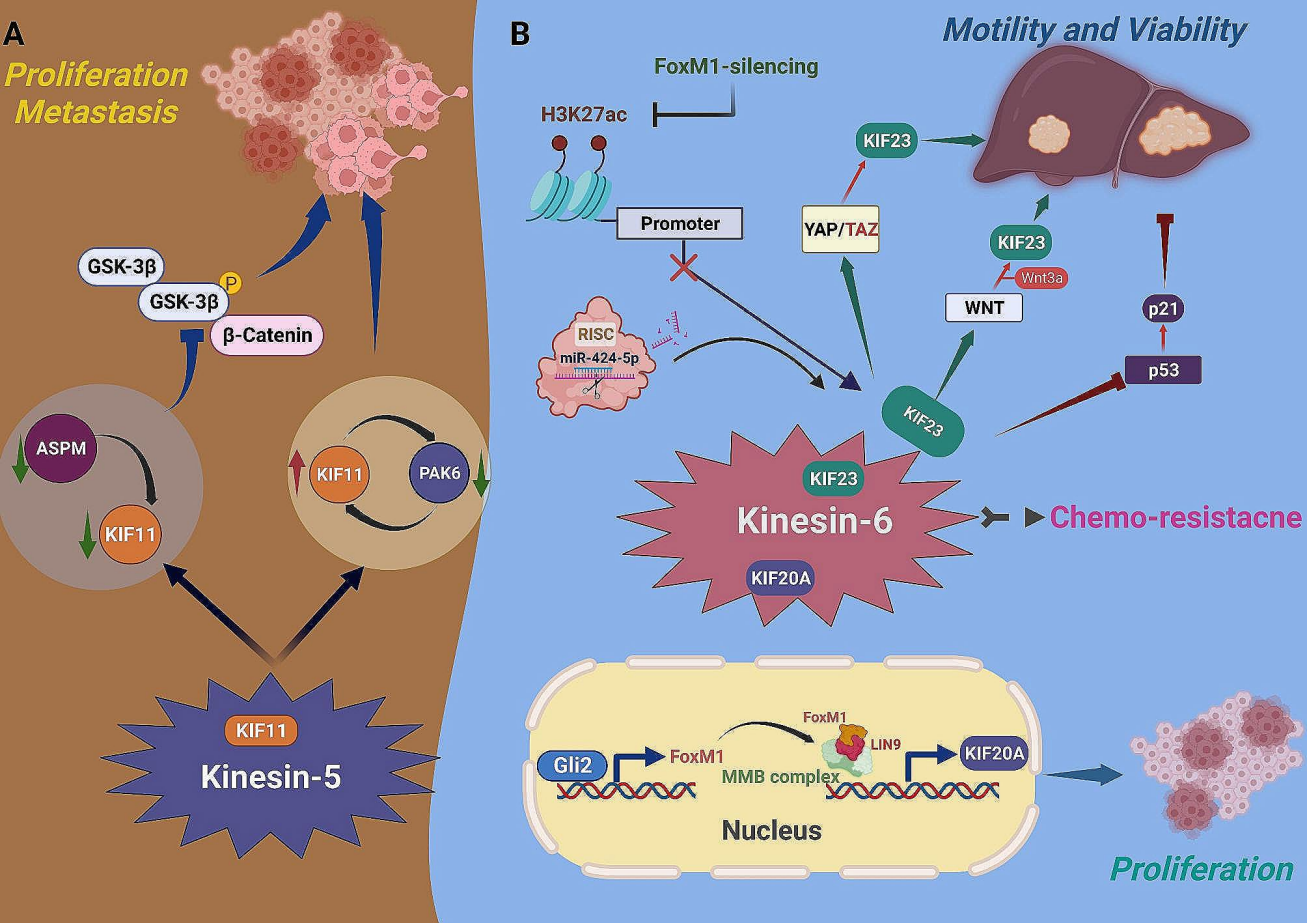
The roles of the kinesin-5, 6 family members in HCC. (**A**) Kinesin-5 family: a reciprocal feedback loop exists between KIF11 and PAK6 and impacts tumor proliferation, invasion, and migration, both PAK6-inhibition or KIF11-overexpression can enhance tumor cells motility and viability. Similarly, the abnormal spindle-like microcephaly-associated protein (ASPM) also negatively modulates KIF11 expression, and the regulatory effects of the ASPM/KIF11 signaling on tumor proliferation, invasion, and migration may be linked to WNT/β-catenin signaling transduction. (**B**) Kinesin-6 family: For KIF20A, identified as an effector in the Hedgehog (Hh) signaling that could be indirectly transcriptional up-regulated by Gli2, acting via FOXM1. For KIF23, exerted as an up- or downstream regulator of three major axes including YAP/TAZ, p53, and WNT/β-catenin signaling pathways with an impact on HCC. Besides, KIF23 could also be recognized and repressed by miR-424-5p, or inhibited by FOXM1 silencing, which was attributed to reduced acetylation level of histone H3 lysine 27 (H3K27ac) at the promoter of KIF23

Similar to the kinesin-2 family, as the only member of the kinesin-5 subfamily, KIF11 has been extensively investigated for its latent significance as a diagnostic and prognostic biomarker in HCC [71–79]. While, besides the bioinformatic-based explorations, the underlying role of KIF11 in HCC progression revealed by experimental observations was also mainly concentrated on its pro-tumoral efficacy. For example, aberrant KIF11 expression has been associated with the facilitation of tumor proliferation, invasion, and migration [80–82]. Mechanically, given the interaction between KIF11 and PAK6 revealed by co-immunoprecipitation (co-IP) assay, as well as the intrinsic reciprocal feedback loop, it was observed that the promotive effects of PAK6-inhibition in proliferation and migration capacities of tumor cells could be reversed by KIF11 knockdown [82]. Similarly, an interactive association between KIF11 and abnormal spindle-like microcephaly-associated protein (ASPM) was also validated by co-IP test, and further experiments found that ASPM inhibition significantly reduced KIF11 expression, thereby suppressing tumor cells proliferation, invasion, and migration in vitro. Theoretically, the ASPM/KIF11 binary complex might participate in the regulation of WNT/β-catenin signaling, since the expression of several pivotal pathway proteins like β-catenin and p-GSK-3β was lessened coming with ASPM-inhibition, and these reductions could be partially reversed by KIF11 overexpression, suggesting the important moderating effect of KIF11 in WNT/β-catenin signaling transduction [80] (Fig. 3A).

### Kinesin-6 family

Regarding the kinesin-6 family, the driving effects of its members (KIF20A, KIF20B, and KIF23) in HCC have also been illuminated in numerous studies. For KIF20A, identified as an effector in the Hedgehog (Hh) signaling, could be transcriptionally up-regulated directly by the FOXM1/MMB complex, and in this process, the Gli2 (an essential regulator in the Hh signaling) was responsible for inducing transcription of FOXM1. Thus, FOXM1 acted as an important ligament in the regulative association between Gli2 and KIF20A, and interrupting this indirect Gli2/KIF20A modulation axis in the Hh signaling might be a suitable therapeutic strategy for HCC [83]. Except for the impact on proliferative vitality of tumor cells induced by KIF20A, the influence on in vitro chemosensitivity was also observed that KIF20A-silencing drastically increased sensitivity of tumor cells to cisplatin and sorafenib, while the intrinsic mechanism remained to be explored [84].

As for KIF23, was illustrated to exert as the upstream regulator or downstream reactor of three major signaling axes in the initiation and progression of HCC, including YAP/TAZ, P53, as well as WNT/β-catenin pathways [85–87]. In the YAP/TAZ signaling, TAZ but not YAP acted as the much more crucial and robust driver in HCC carcinogenesis. By using the CRISPR-interference screening method in transgenic mouse models of primary liver cancer, KIF23 was determined as the key downstream target of TAZ, and reduced KIF23 expression markedly inhibited tumor initiation of HCC models, indicating the attractive significance to exploit pertinent inhibitors or relevant chemo-modifications targeting KIF23 in HCC treatment [87]. Moreover, a previous study also carried KIF23 into WNT signaling that the expression of KIF23 was found to be positively regulated by WNT signaling during Wnt3a treatment, and the research also bound KIF23 as WNT target to the early recurrence and metastasis of HCC, KIF23-silencing effectively inhibited tumor cells proliferation and migration in vitro [86]. Furthermore, as the upstream regulatory element, KIF23 could also damage the activation of the tumor repressive p53 pathway, accompanied by the reductive levels of p21 as well as the enhancement of HCC cells motility and viability in vitro [85].

In addition to the interaction between KIF23 and several important signaling axes mentioned above, other KIF23 actuators or suppressors were also preliminarily explored. For example, the transcriptional activator FOXM1 could epigenetically modify KIF23 expression by influencing the activity of RNA polymerase II (RNA pol II) and acetylation of histone H3 lysine 27 (H3K27ac) at the KIF23 promoter [88]. In addition, miR-424-5p was also thought to recognize and negatively modulate the expression of KIF23, played an inhibitory role in HCC pathogenesis [89]. As with KIF20A, KIF23 was also reported to influence sensitivity to cisplatin and sorafenib [51, 88, 89], indicating that either of KIF20A or KIF23, or say this kinesin subfamily might act an essential role in the maintenance of HCC chemo-resistance (Fig. 3B).

### Kinesin-7 family

Interestingly, by contrast with other KIFs, the only kinesin-7 family member KIF10 (namely CENP-E), seemed to exert an anti-tumor effect on HCC. Decreased CENP-E expression was noticed in HCC tissues and cells compared with normal controls [90, 91], targeted elimination of CENP-E markedly stimulated in vitro tumor proliferation and metastasis, as well as in vivo tumorigenesis [90].

### Kinesin-8 family

As for kinesin-8 family members, KIF18A was identified as an underlying prognostic biomarker exploited via bioinformatic analyses in multiple studies and was demonstrated to be closely associated with HCC malignancy [73, 92–94]. KIF18A-mediated HCC promotion might be correlated with several abnormally activated signaling pathways, including the Akt/MMP7/9 axis to stimulate tumor invasion and migration, and the CyclinB1-related axis to promote proliferation [95].

KIF18B, an important isoform of KIF18A in the kinesin-8 family, also actuates adverse progression of HCC. On the one hand, KIF18B was identified as a biomarker for HCC prognostic evaluation and immunotherapeutic response prediction via bioinformatic-based investigations, the up-regulated KIF18B mRNA expression was demonstrated to predict advanced TNM stage and elevated lymph node metastatic risk of HCC patients [96, 97]. On the other hand, the promotive effects mediated by KIF18B in tumor migration and proliferation might be exerted via the WNT/β-catenin signaling axis, given the corresponding changes of downstream pathway proteins following KIF18B interference [98].

### Kinesin-11 family

**Fig. 4:**
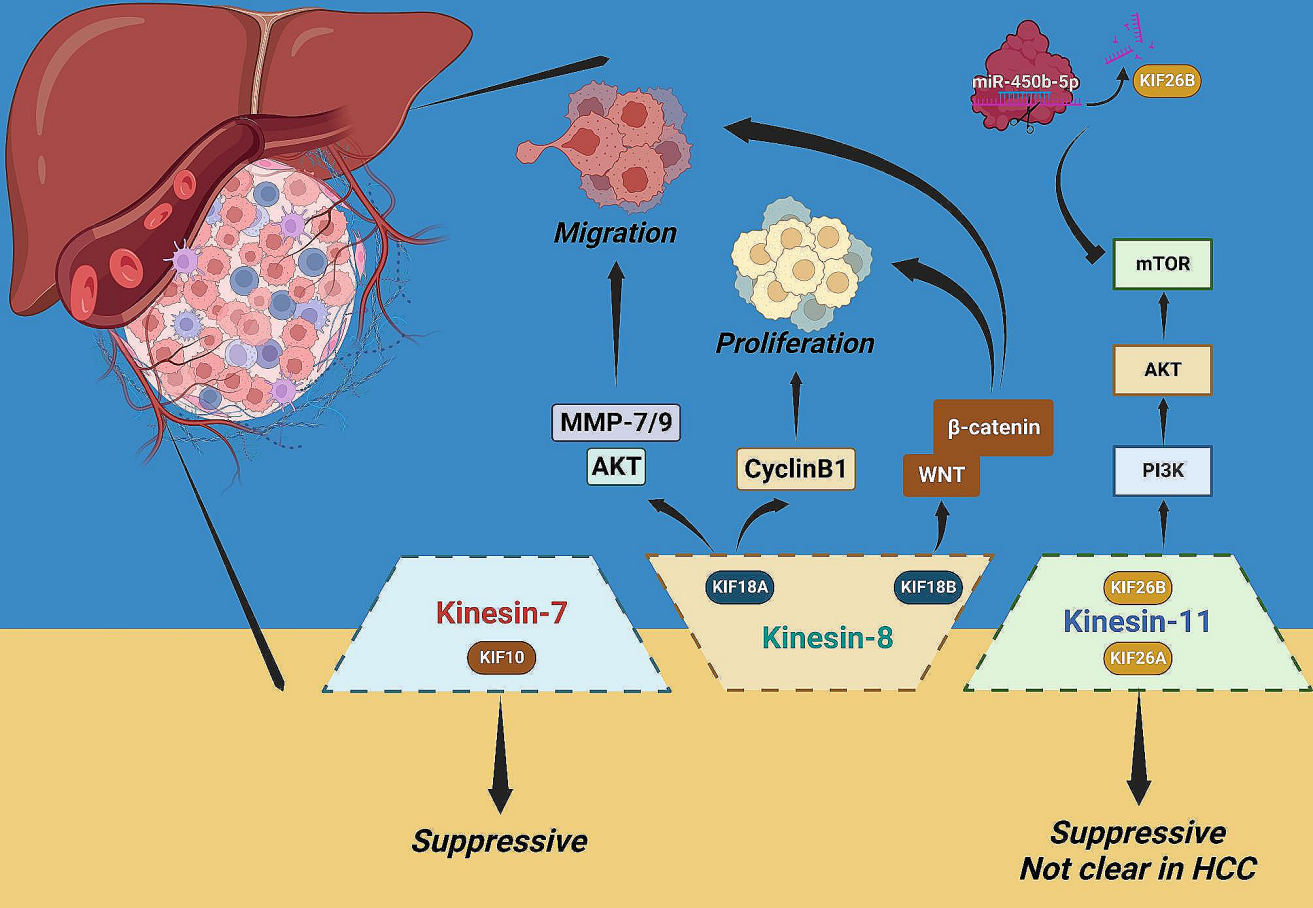
The roles of the kinesin-7, 8, 11 family members in HCC

Interestingly but notably, two members (KIF26A and KIF26B) of this subfamily have opposing effects on tumor progression [99, 100]. Although the fact that KIF26B mediated tumor promotion has been validated in multiple human cancers [34, 99, 101], little is known about its impact on HCC. So far, there was only one research demonstrated that KIF26B participated in PI3K/Akt signaling regulation in HCC, silencing of KIF26B inhibited PI3K/Akt activation by reducing expression of mTOR, p-PI3K, and p-Akt, thereby suppressing the malignant properties of cancer cells. Meanwhile, miR-450b-5p, identified as the executioner of KIF26B, was responsible for the negative adjustment of the above process [102]. Obviously, further investigations around KIF26B, as well as the same family member KIF26A, remain necessary to clarify their impacts on HCC (Fig. 4).

Kinesin-7 family: KIF10 played a suppressive role in HCC proliferation and metastasis.

Kinesin-8 family: KIF18A affected Akt/MMP7/9 signaling with an impact on tumor invasion and migration and CyclinB1-related signaling with an influence on proliferation. KIF18B-mediated tumor promotion was associated with aberrant WNT/β-catenin signaling.

Kinesin-11 family: KIF26A and KIF26B appear to have opposing roles in tumor progression. KIF26B facilitated tumor development via the PI3K/Akt/mTOR signaling pathway and could be negatively regulated by miR-450b-5p. KIF26A restrained progression of many tumor-types, although its impact on HCC remains unclear.

### Kinesin-12 family

**Fig. 5:**
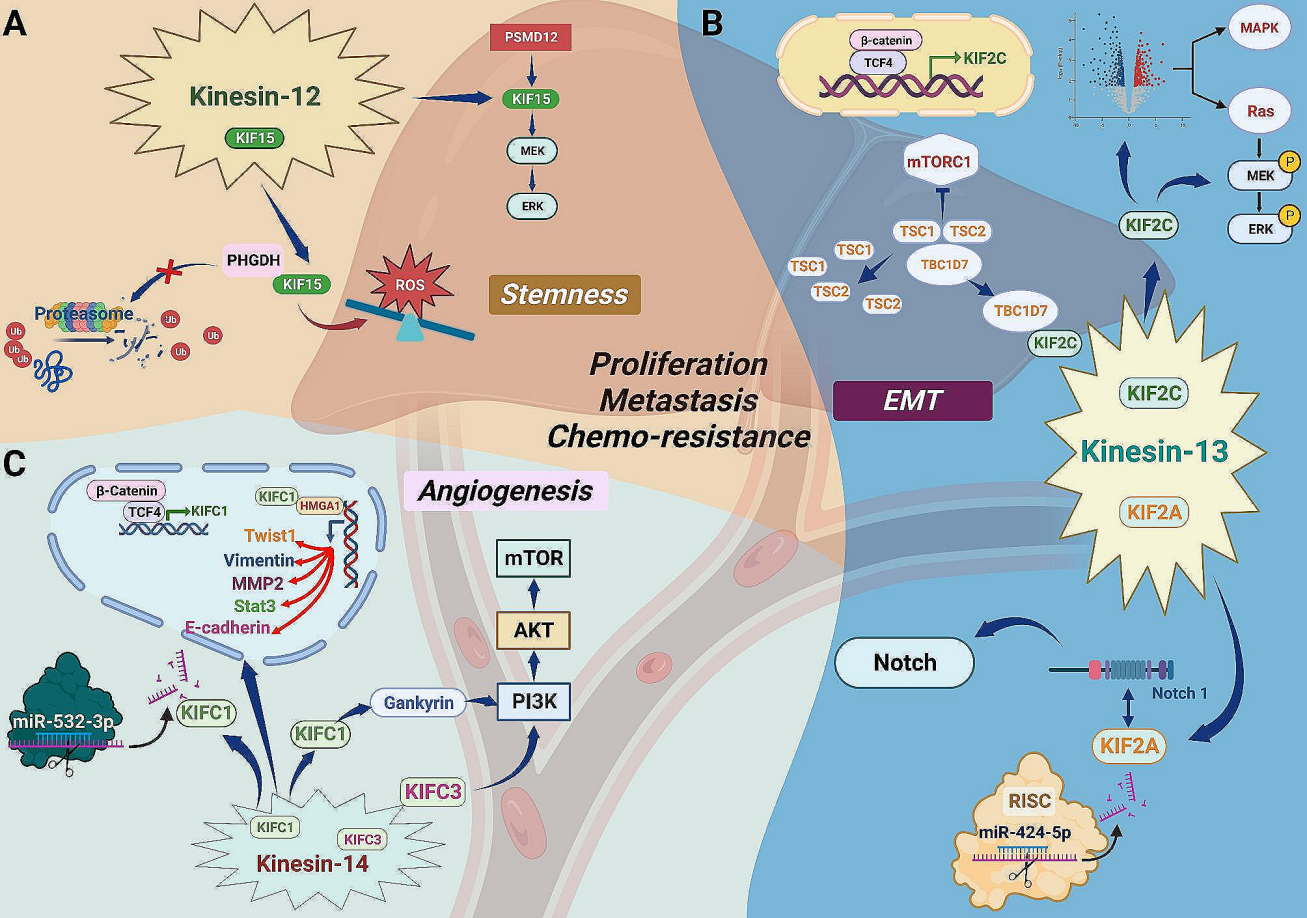
The roles of the kinesin-12, 13, 14 family members in HCC. (**A**) Kinesin-12 family: KIF15 interacts with proteasome 26 S subunit, non-ATPase 12 (PSMD12) and is stimulated by it. And the regulative impacts of KIF15 on HCC cells growth, proliferation, migration, and chemo-resistance have been linked to the MEK/ERK signaling. Furthermore, KIF15 also eliminates intracellular reactive oxygen species (ROS) accumulation and facilitates tumor stem cell phenotype by interacting with phosphoglycerate dehydrogenase (PHGDH) and stabilizing its expression via inhibiting proteasomal degradation. (**B**) Kinesin-13 family: KIF2C mediated cross-talk between WNT/β-catenin and mTORC1 signaling: WNT/β-catenin signaling caused transcriptional up-regulation of KIF2C when TCF4 bound to the KIF2C promoter. Subsequently, KIF2C interacted with TBC1D7 causing destabilization of TSC1/2 and reduced the anti-oncogenic effect of TSC2 on mTORC1 signaling transduction, finally promoted tumor development. Moreover, the pro-tumor impacts of KIF2C might also be attributed to its latent regulatory function in the Ras/MAPK and MEK/ERK signaling axes. Another family member KIF2A, negatively regulated by miR-424-5p, has also been demonstrated to facilitate malignant progression via the Notch 1 signaling, in which KIF2A interacts with Notch 1 and positively regulates its expression. (**C**) Kinesin-14 family: Similar to KIF2C, KIFC1 can also be transcriptionally up-regulated by TCF4, and aberrant activation of KIFC1 facilitates HCC progression through enhancing the transcriptional activity of High Mobility Group AT-Hook 1 (HMGA1), which regulates several cancer-related genes transcription (including STAT3, MMP2, E-cadherin, Vimentin, Twist1) by binding to their corresponding promoter regions. Besides, KIFC1 also participates in the stimulation of PI3K/Akt signaling via up-regulating the expression of gankyrin. Similarly, miR-532-3p plays an inhibitory role in this process by directly suppressing KIFC1 expression. As for KIFC3, it has also been demonstrated to stimulate HCC development through activating PI3K/Akt/mTOR signaling pathway

And regarding to kinesin-12 family, one well-explored member KIF15 was shown to be an impeller in HCC malignant progression (Fig. 5A). In detail, KIF15 interacted with proteasome 26 S subunit, non-ATPase 12 (PSMD12) and was positively regulated as its response gene, which in turn modulated malignant cell behaviors via activating the MEK/ERK signaling [103]. Besides, KIF15 was also demonstrated to be connected with tumor stem cell properties, KIF15-silencing suppressed transcription and translation of stemness-related markers and drastically inhibited spheroid formation as well as growth of HCC patient-derived three-dimensional organoids in vitro. Meanwhile, KIF15 knockdown could also restrain tumor proliferation and migration and enhance sensitivity to chemotherapeutic agents like staurosporine, 5-FU, and cisplatin. Mechanically speaking, KIF15 was found to interact with phosphoglycerate dehydrogenase (PHGDH) and stabilize its expression by reducing the proteasomal degradation, the latter was tightly associated with intracellular reactive oxygen species (ROS) production. KIF15 up-regulation increased the expression of PHGDH, with the effects of eliminating ROS accumulation and facilitating stem cell phenotype and tumor development. Together, the KIF15/PHGDH mediated imbalance of ROS homeostasis was essential to the induction of stem cell property and malignancy, targeting KIF15 could be a promising therapeutic strategy for HCC [104].

### Kinesin-13 family

There are two investigated kinesin-13 subfamily members in HCC, KIF2A and KIF2C (Fig. 5B). For KIF2C, showed elevated expression in HCC specimens and multiple hepatoma cells, in which the protein facilitated proliferation and metastasis both in vitro and in vivo. Theoretically, a potential interaction between KIF2C and Tre2-Bub2-Cdc16 (TBC) 1 domain family member 7 (TBC1D7) was identified by liquid chromatograph mass spectrometry (LC-MS), and this physical association was further validated via the exogenous and endogenous co-IP, substantiating that KIF2C and TBC1D7 could constitute a compound automatically and exert an oncogenic efficiency in HCC. Subsequent more incisive exploration found that the KIF2C/TBC1D7 complex could destroy the interaction between TBC1D7 and TSC1, since both TBC1D7, TSC1, and TSC2 were essential components of the tuberous sclerosis complex (TSC) that the abnormal expression of KIF2C might affect the formation of this ternary complex [105]. To verify the hypothesis, the co-IP assays between TBC1D7 and TSC1/2 were performed under different KIF2C expression conditions. The results indicated that the interaction between TBC1D7 and TSC1/2 was enhanced by KIF2C-silencing and inverted by addition of exogenous KIF2C, implying that KIF2C caused destabilization of the TSC complex with implications for abnormal KIF2C expression. And for the TSC complex, it was reported to suppress activation of the mTORC1 signaling axis depending on complex stability [106]. Therefore, KIF2C knockdown allowed assembly of the ternary complex which inhibited oncogenic mTORC1 signaling, shown by reduced phosphorylation levels of the mTOR and downstream kinases, whereas over-expression of KIF2C had the opposing effect. Furthermore, follow-up investigations determined that the expression of KIF2C could be transcriptionally up-regulated by WNT/β-catenin signaling involving binding of TCF4 to the KIF2C promoter. Taken together, KIF2C appeared to mediate the cross-talk between WNT/β-catenin and mTORC1 signaling. Abnormal WNT/β-catenin signaling mediated the activation of KIF2C, which subsequently antagonized TBC1D7 and destabilized the TSC complex, resulting in the abolishment of TSC2 suppressive efficiency on mTORC1 signaling transduction, thereby activating the mTORC1 signaling and promoting the development of HCC [107]. Besides the annectant role of KIF2C between the above two signaling axes, it was also reported that the tumor-promoting actions of KIF2C might be attributed to the potential regulatory function in Ras/MAPK signaling pathway, which was disclosed mainly according to the transcriptome sequencing differentiation between KIF2C-knockdown and normal control groups, hence the precise modulation mechanism remained to be further explored [108]. Moreover, abnormally activated MEK/ERK signaling participates in promoting cancer cells proliferation and migration, experiments with the MEK/ERK inhibitor U0126 and activator PAF have illuminated this situation. Indeed, the tumor promoting effects could be abrogated by KIF2C-silencing and restored by addition of exogenous PAF, indicating a possible regulatory role of KIF2C in MEK/ERK signaling transduction [109].

Except for KIF2C, another family member KIF2A, has been shown to be negatively regulated by miR-424-5p [110], and the protein was also proved to facilitate viability and motility of HCC cells, as well as tumor angiogenesis via Notch 1 signaling axis, in which KIF2A could interact with Notch 1 and positively induce its expression, thereby enhancing the downstream signaling transduction and HCC promotion [111].

### Kinesin-14 family

Similar to KIF2C, KIFC1 was also determined to be a downstream target of the WNT/β-catenin axis and was transcriptionally up-regulated by binding with TCF4. Once KIFC1 activated abnormally, it was able to promote the epithelial-mesenchymal transition (EMT), chemo-resistance, and proliferation of hepatoma cells, thereby facilitating HCC progression both in vitro and in vivo. On the contrary, KIFC1-depletion could attenuate these stimulative impacts and enhance paclitaxel sensitivity, thus suppressing tumor development. In this process, High Mobility Group AT-Hook 1 (HMGA1), also known as an oncogenic transcription factor that participated in the regulation of multiple cancer-related genes (such as STAT3, MMP2, E-cadherin, Vimentin, and Twist1) transcription via binding to their promoter regions, was identified as an interactor of KIFC1 by co-IP assay. The transcriptional activity of HMGA1 could be stimulated by the transfection of ectopic KIFC1, implying that HMGA1 might be necessary for promotion of HCC by KIFC1. Therefore, KIFC1 serves as an important intermediate signal molecule, plays a crucial part in the modulation of WNT/β-catenin/HMGA1 signaling in HCC pathogenesis [112]. Apart from WNT/β-catenin axis, KIFC1 was also proved to be associated with the regulation of Akt signaling transduction, in which KIFC1 increased expression of gankyrin, resulting in elevated p-Akt and downstream transcriptional factor Twist1, thereby enhancing the invasion ability of HCC cells by actuating the EMT. Meanwhile, the research also pointed out a negative regulatory relationship between KIFC1 and miR-532-3p, resulting from the suppression of KIFC1 expression by miR-532-3p and reduced gankyrin/Akt/Twist1 activity, thus preventing the progression of HCC [113].

As for KIFC3, another kinesin-14 family member, was reported to promote HCC development via the PI3K/Akt/mTOR signaling axis, and the stimulative action of KIFC3 could be suppressed by addition of LY294002 (a highly selective inhibitor targeting PI3K), and combining with KIFC3-depletion and LY294002 treatment displayed a synergistic inhibitory effect, suggesting the potential therapeutic value of KIFC3 in HCC [114] (Fig. 5C).

Taken together, the foregoing summarizes the state of knowledge concerning KIFs and their impacts on HCC carcinogenesis. And it is noteworthy that although numerous relevant investigations have been carried out in HCC, there are still many unknowns that are urgently needed to be further explored, which may be necessary for the exploitation of KIFs-targeted therapeutic tactics in HCC in the future.

## The roles of KIFs in biliary tract carcinomas

In this section, we have shifted our perspective from HCC to biliary tract carcinomas (BTCs), including CCA and GBC, and continue to gather intrinsic regulatory mechanisms of action of KIFs in tumor progression.

First for the CCA, the plurality of existing research mainly focuses on the latent significance of KIFs in tumor prognosis assessment, which we will retrospect later in this work, while valuable investigation about the oncogenic pathway that KIFs dominated or participated in CCA development remains insufficient. One of the few studies has identified KIF1Bβ as a tumor suppressor of CCA with a pro-apoptotic effect exerted via the USP9X/EGLN3 signaling axis [115].

**Fig. 6:**
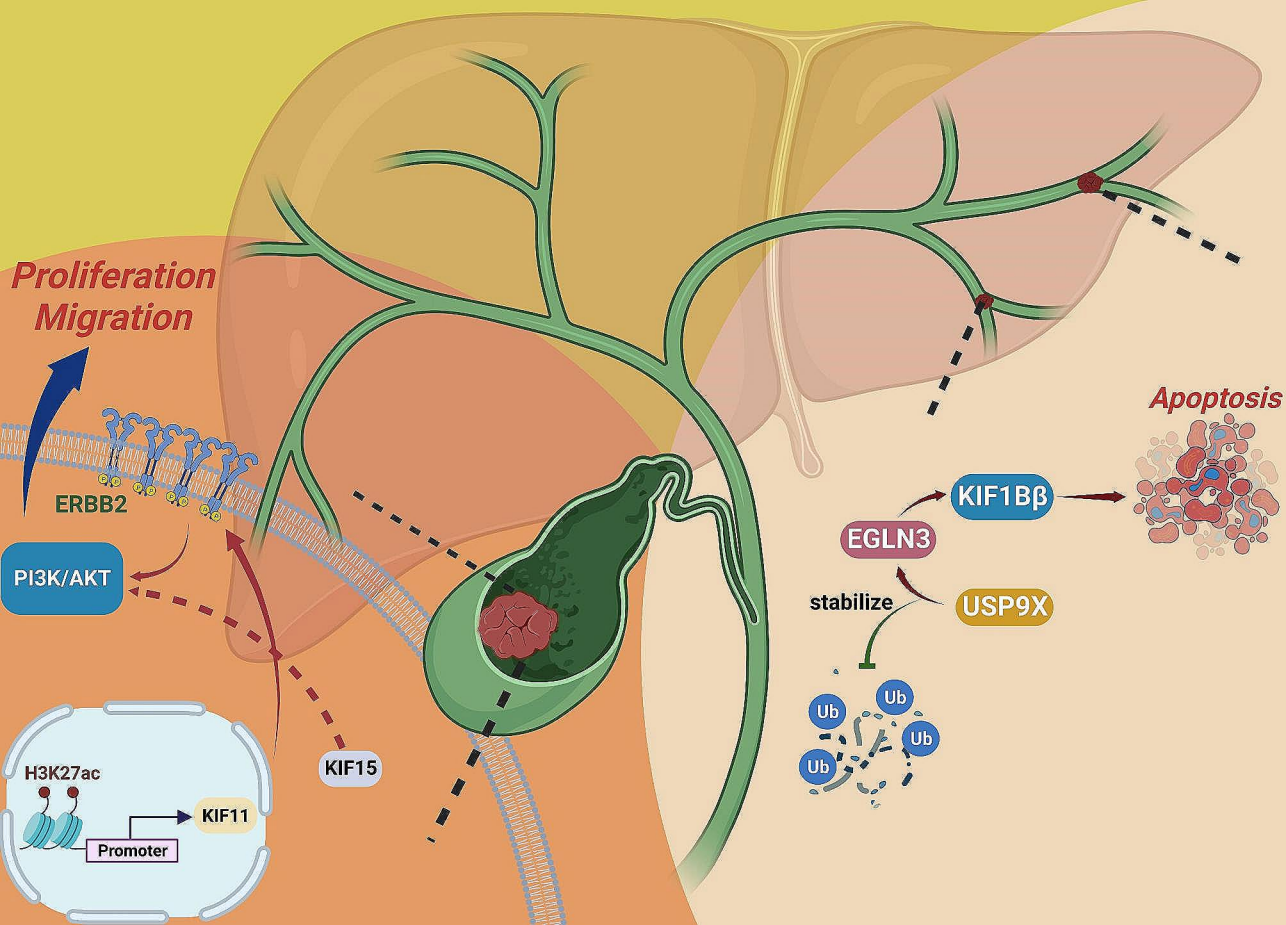
The roles of the kinesin members in biliary tract carcinomas (BTCs)

Regarding GBC, KIF11 transfection was determined to be capable of facilitating tumor growth via an effect on the ERBB2/PI3K/Akt signaling pathway. KIF11-targeted intervention or its specific inhibitor Monastrol treatment, could strikingly induce G2/M cell cycle arrest and suppress tumor growth both in vitro and in vivo. Adversely, ectopic KIF11 expression exerted a promotive effect on the regulation of tumor growth. Mechanically, both KIF11-silencing and Monastrol treatment in GBC cells resulted in the reduction of the ERBB2/PI3K/Akt signaling, an influence could be reversed by ERBB2 overexpression. Moreover, aiming to discover the potential mechanism of aberrant KIF11 expression in GBC, through the combination of bioinformatic analysis and in vitro validation, the research further demonstrated that KIF11 was adjusted via the histone acetylation modification that a markedly abundant H3K27ac level was observed in the promoter area of KIF11, and increased H3K27ac level accounted for elevated KIF11 expression in GBC, which could be stimulated or restrained by addition of acetyltransferase P300 and its specific inhibitor C646. Thus, epigenetic targeting of KIF11 expression may indicate a promising therapeutic strategy for GBC [116]. Additionally, the kinesin-12 subfamily member KIF15 was also proved to be associated with tumor growth and metastasis, in vitro functional experiments demonstrated that KIF15-deficiency blocked tumor cells proliferation and migration, and these findings were robustly supported by the consistent results in vivo. Meanwhile, subsequent mechanistic investigation identified that KIF15 might act on the classical PI3K/Akt signaling pathway to promote GBC development since treatment with an Akt activator could attenuate the anti-tumor impact induced by KIF15-deficiency [117] (Fig. 6).

In cholangiocarcinoma (CCA), the tumor suppressor KIF1Bβ induced apoptosis and was up-regulated by Egl nine homolog 3 (EGLN3), and ubiquitin-specific peptidase 9X (USP9X) acted to de-ubiquitinate and stabilize EGLN3 thereby accelerating this process. In gallbladder cancer (GBC), KIF11 facilitated tumor growth via the ERBB2/PI3K/Akt signaling pathway, and the histone acetylation modification (H3K27ac) positively modulated KIF11 expression. In addition, KIF15 might also have affected the classical PI3K/Akt signaling to promote tumor growth.

In sum, it’s regretful that the involvement of KIFs in BTCs has received little attention hitherto, suggesting that it is both promising and challenging to concentrate on the detailed role of KIFs in BTCs in future research.

## KIFs in HCC and BTCs prognosis

Due to the aberrant dysfunction of KIFs in the development of HBCs, investigations about the prognostic value of KIFs also appeared particularly necessary in clinical practice. Especially for HCC, numerous studies have revealed a close association between KIFs expression and clinical outcomes of HCC patients, indicating the potential significance of KIFs in the prognosis evaluation of HCC. Therefore, the current prognostic evidence of KIFs in HCC and BTCs will be summarized in the coming, respectively.

### KIFs in HCC prognosis

For example, with the results of KIFC1 immunohistochemistry (IHC) staining performed with tumor tissues, 82 HCC samples were separated into low and high KIFC1 expression subgroups, further clinicopathological correlation analysis disclosed that high KIFC1 expression usually signified patients’ poor overall survival (OS) and relapse-free survival (RFS), as well as worse tumor size and nodes status, confirming the oncogenic function and prognostic potential of KIFC1 in HCC [118]. The similar results were ulteriorly verified by IHC staining performed with 168 tumor tissue slices and 30 paired non-cancerous samples, indicating that there was a positive correlation between KIFC1 expression and patients’ poor OS, disease-free survival (DFS), as well as advanced tumor stage, tumor size, and recurrence rate [112]. In addition, a study of 91 paired HCC specimens further demonstrated up-regulation of KIFC1 in tumor tissues, which was also observed to be markedly associated with several clinical features, like cancer embolus, status of relapse and metastasis, as well as tumor-free survival (TFS) time of HCC patients [119]. Analogously, an additional study of 101 HCC specimens, classified into different subgroups by RT-qPCR measurement of KIFC1 expression, linked high KIFC1 expression to several frustrating clinical parameters, like large tumor size, terrible differentiation, high risk of metastasis, as well as poor OS [113]. In sum, the above-mentioned consequences have provided a strong basis for the potential meaning of KIFC1 in prognosis evaluation in HCC.

The expression of KIF2C both in mRNA and protein levels was validated to be obviously elevated in HCC. IHC staining of KIF2C in tumors and matched liver specimens from 149 HCC patients allowed separation into high- and low-KIF2C subgroups. Subsequent Kaplan-Meier (K-M) survival analysis illustrated that patients with higher KIF2C expression suffered a worse OS and DFS, in addition to poorer tumor differentiation and higher relapse risk [107].

According to the IHC intensity of KIFC3 in 36 paired HCC and adjacent normal samples, the abundance of KIFC3 was observed to be up-regulated in tumors with negative consequences for OS of patients with HCC [114].

Additionally, 196 HCC patients who had received hepatic surgery were collected in another research, and the expression levels of KIF2A were analyzed by IHC staining and represented with respective IHC scores. The results discovered that compared with adjacent tissues, the staining intensity of KIF2A showed marked elevation in tumor tissues. K-M survival curves also illuminated that patients at higher KIF2A expression were correlated with poorer OS, and high KIF2A expression was subsequently determined to be an independent hazard factor for poor survival outcomes via the univariate and multivariate cox analyses. Meanwhile, by combining with corresponding clinicopathological features of HCC patients, the research further noted that KIF2A expression was also closely connected with performance status (PS) score, tumor nodule number, tumor dimension, BCLC stage, as well as laboratory indices like AST, AFP, and CA199, implying the intrinsic worth of KIF2A in diagnosis and prognosis evaluation of HCC patients [120].

Furthermore, based on the relative expression of KIF3B evaluated by IHC in 57 paired HCC specimens, a positive association was discovered between KIF3B expression and tumor size, levels of serum AFP, as well as proliferation biomarker ki-67, and a negative association with histological differentiation grade. Moreover, patients with high KIF3B expression displayed an obviously shorter OS than those with low expression. Similarly, KIF3B was also determined to be an independent prognostic predictor for patients with HCC [49].

For KIF4A, the mRNA expression of which was found to be distinctly ascended in an independent HCC cohort of 67 cancerous and paired adjacent tissues. Patients with high KIF4A expression were often linked to poor clinical outcomes, including worse OS, higher risk of recurrence and metastasis, increased tumor size, advanced T stage, as well as terrible nodule status [69]. Meanwhile, according to the IHC staining results of 136 paired tumor pathological sections, it was also demonstrated that high KIF4A expression was positively correlated with poor OS and DFS of HCC patients [66]. Analogously, IHC staining of KIF4A measured with 211 paired HCC tissues showed that the expression of KIF4A was up-regulated and closely associated with poor OS, DFS, vascular invasion, as well as advanced histological grade and TNM stage [67]. All these findings furnished a strong basis for the prognostic significance of KIF4A in HCC.

A retrospective study incorporated 108 primary HCC specimens observed that the expression of Eg5 (KIF11) was about 8.3-fold elevated in tumor tissues compared to non-cancerous ones, and the relative Eg5 expression was demonstrated to be capable of indicating survival results of HCC patients, that patients with higher Eg5 expression were inclined to suffer worse OS and DFS. Indeed, the median OS and DFS of patients with high Eg5 expression were 57.7 and 39.4 months, respectively, which were visibly worse than those at low expression represented by 155.6 months in OS and 126.3 months in DFS [121]. Similarly, by analyzing the mRNA expression of Eg5 in 26 HCC specimens and paired para-cancerous samples, as well as the relative protein expression in 156 HCC specimens, it was also revealed that Eg5 was obviously elevated in tumors, and patients’ OS, liver cirrhosis, and TNM stage were tightly correlated with the expression of Eg5, which further enhanced the predictive performance of Eg5 in prognosis evaluation of patients with HCC [122].

Moreover, both the mRNA and protein levels of KIF14 measured by RT-qPCR and Western blotting were observed to be ascended in tumors compared to peritumoral samples. Subsequent K-M survival analysis of 102 HCC patients associated high KIF14 expression with poor DFS and late tumor grade and stage, indicating the potential prognostic value of KIF14 in HCC [52].

Additionally, the relative KIF15 mRNA expression levels measured by RT-qPCR with 59 cancerous samples and 49 matched peritumoral samples also indicated that high KIF15 expression was independently connected with poorer OS of HCC patients, and was also significantly associated with undesirable pathological parameters, including larger tumor size and inferior differentiation [123]. Meanwhile, the mRNA levels of KIF15 detected in another HCC cohort incorporating 120 tumoral and paired non-cancerous tissues were shown to be significantly overexpressed, which was also demonstrated to be independently correlated with worse OS and increased risk of recurrence, as well as a close relationship with several clinicopathological parameters, including tumor size, single or multiple, TNM stage, vascular invasion, and encapsulation status [104].

KIF18A, with elevated mRNA expression in 216 HCC samples paired with para-carcinoma tissues measured by RT-qPCR, was found to be significantly correlated with serum AFP, tumor size, TNM stage, and portal vein tumor thrombus (PVTT), indicating the possible efficiency of KIF18A in HCC diagnosis. K-M survival curves further illustrated a negative correlation between KIF18A expression and patients’ OS and DFS that patients at low KIF18A expression showed better survival outcomes compared to those at higher expression (mean OS: 55.06 months vs. 39.26 months; mean DFS: 50.14 months vs. 30.84 months), suggesting the independent predictive value of KIF18A in HCC prognosis evaluation [124].

In the HCC cohort of a total of 210 samples (172 in pairs and 38 individual tumor specimens), the aberrant high expression of KIF20A detected by IHC staining was discovered to be notably connected with tumor grade, TNM stage, and vascular invasion status, which were predictive of reduced OS and DFS for patients with HCC [83].

Additionally, based on the correlation analysis between KIF21B expression and matched pathological features of 186 HCC patients, it was disclosed that high KIF21B expression was significantly associated with vascular invasion, TNM stage, and HBsAg status, indicating the utility of KIF20A as an independent risk factor for poor OS and DFS in patients with HCC [70].

With the IHC measurement of KIF26B in HCC tissue microarrays (TMA) consisting of 93 paired tumor and peritumoral samples, it was observed that the expression of KIF26B was elevated and positively correlated with advanced tumor stage and grade, which could also indicate a shorter OS for HCC patients [102].

And KIF23, had two different splice variants, KIF23 V1 (nucleus localization) and KIF23 V2 (cytoplasm localization), was abnormally expressed in 144 HCC tissues based on the IHC staining consequences. By integrating the corresponding clinical features, it was interesting that there was a negative association between KIF23 V1 expression and patients’ OS that patients with positive KIF23 V1 expression displayed a better 5-year OS than those without the expression of KIF23 V1, implying that it might exert a protective role in the progression of HCC, while no significant connection was observed between KIF23 V2 expression and patients’ survival outcomes [125].

Similarly, unlike most kinesin members, there was a negative correlation between KIF1B protein expression and HCC vein invasion, as well as recurrence status, although no significant association was found for mRNA levels. Unexpectedly, the radios of KIF1B mRNA in 68 HCC and paired normal tissues were demonstrated to be associated with OS and DFS, that down-regulated KIF1B mRNA radios in HCC pairs (higher KIF1B expression in peritumoral samples than in tumors) indicated a worse survival outcome, while there was no obvious correlation with most clinicopathological features in terms of individual KIF1B mRNA expressions. These findings noted the complicated roles of KIF1B in HCC development, despite the seemingly ambiguous relationship between distinct KIF1B expression patterns and some of the clinical parameters, it might prove to be a protective prognostic indicator for patients with HCC [55].

Analogously, by contrast with many other KIFs, CENP-E (KIF10) expression was found to be singularly decreased in HCC specimens. By uniting the IHC staining results of 90 paired HCC samples and their corresponding pathological features, it was further demonstrated that patients with low CENP-E expression were usually accompanied by poor OS, large tumor size, late TNM stage, as well as increased metastasis, suggesting the latent protective role of CENP-E in the development of HCC [90].

Moreover, relying on the sequencing data of independent HCC cohorts acquired from multiple public datasets, including the Cancer Genome Atlas (TCGA), Gene Expression Omnibus (GEO), and International Cancer Genome Consortium (ICGC), and their corresponding clinicopathological information, the prognostic significance of several kinesin family members (KIFC1, KIFC3, KIF2A, KIF2C, KIF3A, KIF4A, KIF4B, KIF10, KIF11, KIF14, KIF15, KIF18B, KIF19, KIF20A, KIF20B, and KIF23) was preliminarily investigated via bioinformatic analysis, relevant findings further supported the potential value of aberrant KIFs expression in prognosis evaluation of patients with HCC [50, 81, 84, 103, 108, 109, 126–135].

### KIFs in BTCs prognosis

As for bile duct carcinomas, including cholangiocarcinoma (CCA) and gallbladder cancer (GBC), the prognostic significance of KIFs has also been investigated. In CCA, based on the proteomic analysis with plasma samples from Opisthorchis viverrini (OV) and N-nitrosodimethylamine induced hamster CCA models, it was indicated that the expression of KIF18A was drastically up-regulated in CCA models compared to the normal control, indicating the potential significance of KIF18A in the early diagnosis of CCA, while the above findings were acquired mainly according to the animal models that large-scale clinical trials remained needed to further verify the diagnostic and prognostic meaning of KIF18A in patients with CCA [136]. In addition, RNA-seq data from the GEO database and IHC analysis of CCA samples have shown elevated KIF4A expression in CCA. Survival analysis of KIF4A based on the GEPIA database further demonstrated that patients with higher KIF4A expression displayed poorer OS, implying the potential prognostic value of KIF4A in CCA [137].

While in GBC, due to the lower morbidity and relatively limited open-access sequencing data, relevant investigations about KIFs in the prognosis evaluation of GBC are still insufficient, and further exploration is needed to clarify the relevance of KIFs in GBC.

Taken together, relevant evidence about prognostic values of KIFs in HBCs has been summarized in Table 1. Spontaneously, for a more integrated illumination of KIFs, their significance in tumor-targeted therapy will be further discussed in the following part.

**Table 1:** Association between KIFs and prognosis of hepatobiliary carcinomas

KIFs	Subtribe	Cancer type	Expression	Data sources	Correlation with prognosis	References
KIFC1	Kinesin-14 A	HCC	Up-regulated	IHC staining performed with 82 tumor samples	High expression indicates poor OS (*p* = 0.011), RFS (*p* = 0.016), and is positively correlated with tumor size (*p* = 0.034) and lymph node status (*p* = 0.040)	[118]
KIFC1	Kinesin-14 A	HCC	Up-regulated	IHC staining of 168 tumor and 30 paired normal liver tissues	High expression correlates with poor survival outcomes (mean survival time: 45.57 months vs. 74.15 months, *p* < 0.002), including poor OS (*p* < 0.001), DFS (*p* < 0.001), and advanced tumor stage (*p* = 0.009), increased tumor size (*p* = 0.031) and recurrence hazard (*p* = 0.037)	[112]
KIFC1	Kinesin-14 A	HCC	Up-regulated	IHC staining of 91 paired HCC samples	Overexpression predicts a shorter TFS (*p* = 0.004), and closely correlates with tumor recurrence (*p* = 0.015) and metastasis (*p* = 0.001)	[119]
KIFC1	Kinesin-14 A	HCC	Up-regulated	RT-qPCR analysis of 101 paired HCC specimens	High expression indicates poor OS (*p* < 0.01), large tumor size (*p* = 0.0281), terrible differentiation (*p* = 0.0289), and elevated metastatic risk (*p* = 0.0085)	[113]
KIF2C	Kinesin-13	HCC	Up-regulated	IHC staining of 149 paired tumor and normal liver tissues	Overexpression predicts poor OS (*p* = 0.002), DFS (*p* = 0.021), and closely associates with tumor differentiation (*p* = 0.016) and recurrence (*p* = 0.025)	[107]
KIFC3	Kinesin-14B	HCC	Up-regulated	IHC staining of 36 paired HCC and adjacent normal samples	High expression correlates negatively with the OS (*p* = 0.037)	[114]
KIF2A	Kinesin-13	HCC	Up-regulated	IHC staining of 196 paired HCC samples	High expression drastically correlates with tumor size (*p* = 0.015), lymph node status (*p* = 0.018), BCLC stage (*p* < 0.001), and independently predicts poor OS (*p* = 0.001)	[120]
KIF3B	Kinesin-2	HCC	Up-regulated	IHC staining of 57 paired HCC specimens	Overexpression indicates poor OS (*p* < 0.010) and late histological grade (*p* < 0.01), and positively correlates with tumor size (*p* = 0.002), serum AFP levels (*p* = 0.041), as well as Ki-67 expression (*p* < 0.01)	[49]
KIF4A	Kinesin-4	HCC	Up-regulated	RT-qPCR analysis of 67 tumor and matched adjacent normal tissues	High expression predicts worse OS (*p* = 0.003), increased risk of recurrence (*p* = 0.0123) and metastasis (*p* = 0.022), as well as increased tumor size (*p* = 0.0143), T stage (*p* < 0.0001), and nodule status (*p* = 0.0029)	[69]
KIF4A	Kinesin-4	HCC	Up-regulated	IHC staining of 136 paired HCC samples	High expression positively correlates with poor OS (*p* < 0.001) and DFS (*p* = 0.0337)	[66]
KIF4A	Kinesin-4	HCC	Up-regulated	IHC staining of 211 paired HCC tissues	Overexpression distinctly correlates with poor OS (*p* = 2.81*10^− 11^), DFS (*p* = 4.99*10^− 9^), histological grade (*p* = 0.001), vascular invasion (*p* = 0.045), and TNM stage (*p* = 4.91*10^− 7^)	[67]
KIF11	Kinesin-5	HCC	Up-regulated	RT-qPCR analysis of 108 paired primary HCC samples	High expression predicts poor OS (median OS: High-57.7 months, Median-75.3 months, Low-155.6 months, *p* = 0.002) and DFS (median DFS: High-39.4 months, Median-46.2 months, Low-126.3 months, *p* = 0.001)	[121]
KIF11	Kinesin-5	HCC	Up-regulated	IHC staining of 156 paired HCC specimens	High expression negatively correlates with the OS (*p* = 2.43*10^− 8^) and TNM stage (*p* = 0.000151)	[122]
KIF14	Kinesin-3	HCC	Up-regulated	RT-qPCR analysis of 102 paired HCC tissues	Overexpression indicates worse DFS (*p* < 0.001), advanced tumor stage (*p* = 0.013) and grade (*p* = 0.002)	[52]
KIF15	Kinesin-12	HCC	Up-regulated	RT-qPCR analysis of 59 tumor and 49 paired peritumoral tissues	High expression independently predicts worse OS (*p* < 0.001), and significantly correlates with larger tumor size and inferior differentiation (*p* < 0.012, *p* < 0.040, respectively)	[123]
KIF15	Kinesin-12	HCC	Up-regulated	RT-qPCR analysis of 120 paired HCC tissues	High expression associates with worse OS (*p* = 0.0004), increased recurrence hazard (*p* = 0.0011), as well as a close relationship with tumor size (*p* = 0.002), TNM stage (*p* < 0.001), vascular invasion (*p* < 0.001), tumor multiplicity (*p* = 0.013) and encapsulation status (*p* = 0.001)	[104]
KIF18A	Kinesin-8	HCC	Up-regulated	RT-qPCR analysis of 216 paired HCC tissues	High expression negatively correlates with the OS (Mean OS: high-39.26 months vs. low-55.06 months; *p* = 0.001) and DFS (Mean DFS: high-30.84 months vs. low-50.14 months; *p* = 0.001), and positively correlates with tumor size (*p* = 0.009), TNM stage (*p* < 0.001), and serum AFP levels (*p* = 0.030)	[124]
KIF20A	Kinesin-6	HCC	Up-regulated	IHC staining of 210 HCC samples (172 in pairs and 38 individual tumor specimens)	Overexpression predicts poor OS (*p* = 7.024*10^− 6^ ) and DFS (*p* = 1.634*10^− 5^), and notably correlates with tumor grade (*p* = 3.919*10^− 5^), TNM stage (*p* = 0.002), and vascular invasion (*p* = 0.005)	[83]
KIF21B	Kinesin-4	HCC	Up-regulated	IHC staining of 186 tumor and matched adjacent normal tissues	High expression predicts worse OS (*p* = 0.0002), DFS (*p* = 0.0005), TNM stage (*p* = 0.046), and vascular invasion (*p* = 0.012)	[70]
KIF26B	Kinesin-11	HCC	Up-regulated	IHC staining of HCC tissue microarrays containing 93 paired tumor samples	High expression indicates poor OS (*p* = 0.0145), and drastically correlates with late tumor stage and grade (*p* = 0.017, *p* = 0.004, respectively)	[102]
KIF23	Kinesin-6	HCC	Up-regulated	IHC staining of 144 HCC tissues	Positive expression of KIF23 V1 predicts better 5-year OS (*p* = 0.0052)	[125]
KIF1B	Kinesin-3	HCC	No significant differences	WB and RT-qPCR analysis of 68 HCC and matched normal tissues	Down-regulation of KIF1B mRNA radios indicates worse OS (Median OS: downregulated-13.5 months vs. upregulated-20.0 months, *p* < 0.05) and DFS (Median DFS: downregulated-11.5 months vs. upregulated-19.5 months, *p* < 0.05)	[55]
KIF10	Kinesin-7	HCC	Down-regulated	IHC staining of 90 paired HCC specimens	Decreased KIF10 expression associates with poor OS (*p* < 0.05), large tumor size (*p* = 0.038), advanced TNM stage (*p* = 0.009), and positive metastasis (*p* = 0.013)	[90]
KIF18A	Kinesin-8	CCA	Up-regulated	Proteomic analysis with plasma samples from induced hamster CCA models	Overexpression contributes to the early diagnosis	[136]
KIF4A	Kinesin-4	CCA	Up-regulated	RNA-seq and survival data from the GEO and GEPIA databases	High expression indicates poor OS (*p* = 0.035)	[137]

## Targeting KIFs in hepatobiliary carcinomas

Uncontrolled cell proliferation represents one of the acknowledged hallmarks of malignancies [138], and rectifying the aberrant cell proliferation is absolutely an attractive anti-tumor therapeutic strategy. Since mitosis refers to a fundamental life process for eukaryotic division and growth, anti-mitotic therapeutic logically becomes the entry point of anti-tumor treatment. Noteworthily, microtubule-targeted agents (MTAs) belong to one of the most representative anti-mitotic drugs that have been widely applied in clinics, particularly in chemotherapy for solid tumors [139–142]. During the mitotic phase, the microtubule kinetic stability (implicating a highly dynamic polymerization/depolymerization process) is critical for proper spindle formation and exact chromosomal segregation. Therefore, disrupting this dynamic property usually contributes to spindle dysfunction, and subsequent inaccurate separation process or mitotic retardation, eventually induces cell death. MTAs precisely exert this efficiency, which are consist of two major classes: one plays a microtubule-stabilization role, represented by taxanes (paclitaxel and docetaxel), and another mediates an opposing function, including vinca alkaloids, colchicine, as well as eribulin [143]. For taxanes, paclitaxel occupies an unassailable position in cytotoxic therapy for various solid tumors, particularly for non-small cell lung cancer (NSCLC), breast or ovarian cancers, and castration-resistant prostate cancer [144, 145]. Theoretically, paclitaxel administers the mitotic-suppression effect via a diverse range of mechanisms. On the one side, the drug is thought to bind the microtubule subunit with the result of stabilizing microtubule aggregation and inhibiting depolymerization, causing grievous destruction on the dynamics and subsequent mitotic arrest and cell death [146]. Interestingly, recent research also notes that paclitaxel-mediated cytotoxicity may depend on incorrect chromosomal segregation or enhanced chromosomal instability, rather than its role in the kinetic process [147, 148]. On the other side, paclitaxel can also impede microtubule-dependent intracellular substance trafficking to exert cytotoxic effects. Like reducing translocation of the androgen receptor (AR) from the cell surface to the nucleus in castration-resistant prostate cancer cells [145, 149–151], and impeding transport of DNA-damage and repair components [152]. In regard to microtubule-destabilizers, just as their name implies, perturb microtubule polymerization and cause mitotic arrest. By binding with distinct tubulin domains, these destabilizers can be further subdivided into vinca-sequence recognizers (represented by vincristine, cryptophycins, dolastatins, and eribulin), and colchicine-region binders (including colchicine and its analogues) [143]. Although some valid practices have already been conducted in multiple human cancers by treating with microtubule destabilizing agents, such as lymphomas, breast, lung, and bladder carcinomas, the drug resistance distinctly limits their applications during clinical trials, making this class of chemotherapeutic agents less reliable than paclitaxel [146]. Moreover, different from the impacts on microtubule dynamics, both above MTAs can also robustly suppress tumor development via targeting tumor vasculature, either restraining neo-angiogenesis or abrogating the integrity of blood vessels. And given the fact that targeting vasculature may produce a more rapid and reversible effect, making those short but quick-acting microtubule destabilizers more efficient than those microtubule-stabilizing drugs that induce mitotic arrest [143].

Furthermore, numerous attempts have been made to refine MTAs, one of the salient representatives is the nanoparticle albumin-bound paclitaxel (nab-paclitaxel), which has been used in combination with gemcitabine as the first choice for advanced pancreatic cancer and breast cancer, especially for metastatic triple-negative breast cancer [153–157]. Nevertheless, the intractable resistant issue (mainly attributed to the drug efflux mediated by ATP binding cassette family members, modifications on microtubule-protein interactions, as well as aberrant anti-apoptosis signaling transduction [158]), and severe toxic side effects, such as peripheral neurotoxicity and marrow toxicity [143], have greatly limited the further development of these MTAs. Thus, there is an acknowledged need for more efficient, highly selective and minimally toxic novel microtubule complex targeted drugs, and kinesins are promising alternative targets due to the critical role of microtubule-binding kinesins in tumor initiation and progression. Here, we will continue to overview the current research and application advancements of kinesin-targeted inhibitors in anti-mitotic therapy of malignancies, especially HBCs.

Among 45 authenticated kinesin family members, kinesin spindle protein (KSP, also known as Eg5 or KIF11) aimed inhibitors have been explored intensively, and some of them have already come into clinical trials. As an N-type kinesin member attached to the kinesin-5 family, Eg5 exerts plus end-directed motor activity and participates in modulating appropriate centrosome segregation and bipolar spindle formation at the beginning of mitosis, thereby ensuring the subsequent proper chromosome separation [159]. Hence, targeting abnormal Eg5 turns out to be a prospective anti-mitotic strategy. Since the emergence of the selective Eg5 inhibitor monastrol (suppressing its driving capacity by specifically inhibiting the ATPase vitality of the motor domain), plenty of practices have been made to investigate other promising Eg5-aimed small molecules to provide more choices for anti-mitotic-based tumor chemotherapy. So far, several other Eg5 inhibitors have been characterized, including monastrol and its chemical analogues: Ispinesib, S-Trityl-L-cysteine (STLC), and Arry-520, which are classified into the loop L5 binding allosteric suppressors, while GSK-1, PVZB1194, and BRD9876, particularly inhibits Eg5 by binding the helix-α4/6 region of the motor domain, categorized as Eg5 ATP-binding rivalrous depressors [160]. Moreover, some natural compounds have also been determined as Eg5-targeted inhibitors. For example, the fungus derivative Terpendole E restrains Eg5 biological activity by binding the loop L5 region, a similar but not identical inhibitory mechanism makes it effective against cells with resistance to certain allosteric inhibitors [161, 162]. Additionally, other natural products such as non-selective kinesin suppressor Adociasulfate-2 (AS-2) [163], sperm-toxic agent Gossypol [164], diterpenoid compound Tonantzitlolone A [165], as well as Curcumin [166], a natural compound extracted from turmeric, are also identified to exert repressive effects on Eg5. In addition to the agents mentioned above, exogenous modulation of Eg5 expression (represented by RNA interference (RNAi) technique) is also a promising therapeutic strategy in clinical practice, like Eg5-silencing induces tumor inhibition mediated by the delivery of nano or cationic liposomes coated siRNAs/shRNAs [167].

As for clinical transformation, plenty of the tested suppressors have already been carried into clinical trials. Although the phased outcomes are not yet satisfactory, some hopeful advantages can also be observed like decreased neurotoxicity compared with taxanes, as well as the potential of Arry-520 (filanesib in clinical) in the treatment of relapsed or intractable multiple myeloma. The relevant clinical trial results are well reviewed in [160, 168], due to space constraints, we will not go into details here. In regard to hepatobiliary carcinomas, observations from the phase II trial of SB-715992 (a KSP-targeted inhibitor) failed to show the superiority of monotherapy for patients with metastatic HCC, although the treatment was well tolerated [169]. And in CCA, based on the non-targeted high-throughput drug screening, another KSP suppressor SB-743921 was identified to exert a prominent anti-tumor activity against multiple human CCA cell lines, cell line-derived xenograft (CDX) immunodeficient mouse models, as well as patient-derived xenograft (PDX) CCA models. And compared with conventional chemotherapy agents like gemcitabine and paclitaxel, SB-743921 displayed a non-inferior tumor suppression, particularly in PDX models. Although the drug combination did not present a stronger inhibitory effect, the current consequences have already determined the restrained role of SB-743921 in CCA, which deserves further attention via clinical trials with CCA patients [170].

In addition to KSP, another kinesin family member CENP-E (or KIF10) has also been carried into clinical translational practices, besides the anti-tumor action exerted by CENP-E inhibitor (GSK923295) in preclinical models [171, 172], the results from an inspiring phase I clinical trial further indicated its potential for treatment of refractory solid tumors [173]. However, other reported CENP-E inhibitors such as UA62784, syntelin, PF-2771, and Compound-A, basically remain in the preclinical phase [174]. And in HCC, the anti-proliferative effect induced by GSK923295 has only so far been demonstrated in CDX and PDX models [175], indicating that there is still plenty of space to explore these CENP-E targeted inhibitors.

Furthermore, several other kinesin inhibitors, such as KIF15-IN-1 (a synergistic inhibitor of Eg5) targeting KIF15, produces an effective suppression on tumor growth by combining with Eg5 inhibitors, which may be expected to overcome chemo-resistance during ispinesib monotherapy [176, 177]. And some relatively infrequent KIFs-suppressors, including pan kinesin-13 members targeted inhibitor DHTP [178], and KIF2C depressor Sulfoquinovosylacylglycerols (SQAGs) [179], KIF20A targeted suppressor paprotrain [180], KIF18A contraposed inhibitor BTB-1 and its synthetic derivatives [181, 182], as well as allosteric inhibitors of KIFC1: AZ82, SR31527, and CW069 [183–185], etc. Regrettably, the verification of the anti-tumor effects of these targets is still in the preclinical stage, and also remain to be investigated for clinical activity against HBCs.

Obviously, KIFs targeted inhibitors are achieving increasing attention, as indicated by the current review. Although we have tried to summarize as comprehensively as possible, what is involved in this paper is only the tip of the iceberg, and many emerging compounds remain under development. And for HBCs, besides the existing practice of Eg5 suppressors (SB-715992 and SB-743921) in tumor therapy, no other relevant targets reported yet, indicating the prodigious exploratory potential towards this direction.

## Conclusion and future perspective

In this paper, we started with the overall landscape of the kinesin family members, to the carcinogenic roles of KIFs in HCC and BTCs, followed by their significance in tumor prognosis evaluation, and eventually returned to the bedside, reviewed the relevant KIFs targeted inhibitors in tumor therapy. Generally, characteristics of kinesin family members, oncogenic roles of KIFs and their diagnostic and prognostic significance in HBCs, as well as KIFs inhibitors in tumor therapy have all been comprehensively addressed in the current review. Meanwhile, compared with traditional anti-microtubule drugs, anti-mitotic therapies based on KIFs are also promising alternatives for tumor treatment with advantages of improving treatment toxicity.

Furthermore, most KIFs-mediated tumor progression is associated with abnormally activated oncogenic signaling pathways. Based on the above discussion, it’s not difficult to find that different KIFs may be involved in the regulation of same signaling transduction and then exert distinct functions. For example, as outlined above, many KIFs are correlated with Akt signaling pathway, like KIF3B, KIF14, KIF4A, KIF18A, KIF26B, KIFC1, and KIFC3 in HCC, as well as KIF11 and KIF15 in GBC. Additionally, there are also several WNT/β-catenin signaling associated KIFs, such as KIF11, KIF23, KIF18B, KIF2C, and KIFC1, and MEK/ERK signaling related KIFs, including KIF15, and KIF2C (**Supplementary Fig. **1). These distinct KIFs modulate various tumor-promotion effects via acting as the upstream driver or downstream effector of specific signaling axis, which means that targeting KIFs combined pathway inhibitors is also expected to be a novel strategy for precision cancer therapeutic, with the possibility to improve the limitation and resistance of mono-therapy, and serving as potential biomarkers to predict susceptibility to pathway inhibitors.

Actually, compared to the treatment with classical anti-mitotic agent paclitaxel, the combination with ipatasertib (an ATP-competitive Akt inhibitor) has been discovered to moderately improve the PFS and OS in patients with triple negative breast cancer (TNBC) [186]. Meanwhile, patients with PIK3CA-mutated metastatic gastric cancer receiving capivasertib (another developed ATP-competitive Akt inhibitor) and paclitaxel treatment also displayed a better objective response rate than those at PI3KCA-unmutated status receiving capivasertib mono-therapy [187]. And treatment with capivasertib plus paclitaxel also contributes to lengthening PFS and OS for patients with TNBC compared to the paclitaxel plus placebo regimen, especially for those carrying PIK3CA/AKT1/PTEN altered tumors [188]. Moreover, the results of several initiated phase III clinical trials evaluating the efficacy of combined paclitaxel and Akt inhibitors in tumor treatment are also expected to further elucidate the feasibility of the combination of anti-mitosis and Akt-inhibition in cancer therapy [189]. These above findings provide a substantial basis for the development of KIFs-targeted anti-mitosis suppressors to synergize with conventional pathway inhibitors in tumor therapy.

In this work, this synergistic enhancement effect has also been observed in HCC. Such as the combination of KIFC3-deleption and LY294002 treatment in PI3K/Akt signaling, and KIF2C-silencing combined with U0126-inhibition in MEK/ERK pathway. Additionally, K858, characterized as the Eg5 inhibitor, constrained the proliferative ability of breast cancer cells through inducing apoptosis, meanwhile, this effect could be attenuated by the up-regulation of survivin induced by K858, and combined with Akt inhibitor wortmannin could reduce the expression of survivin, thereby sensitizing cancer cells to K858 [190]. Furthermore, suppression of ERK1 activity could enhance the sensibility of neuroblastoma cells to CENP-E inhibitor GSK923295, and the combination of MEK/ERK and CENP-E inhibition displayed a drastic synergistic inhibitory effect on the growth of neuroblastoma, pancreatic, and colon cancer cells [191]. However, further clinical translations have not been carried out based on these findings.

Besides, for certain tumors that are resistant to specific pathway inhibitors, selecting KIFs-targeted inhibitors as second-line treatment may be conducive to ameliorating this dilemma. For example, tumor cells with the acquired resistance to tyrosine kinase inhibitors (TKIs) actuated by the activation of YAP/FOXM1 axis showed an up-regulation of spindle assembly checkpoint (SAC) proteins, such as aurora kinase, survivin, and kinesin spindle protein, which mediated these resistant cells more vulnerable to SAC inhibitors [192]. Moreover, increased sensitivity to mitotic disruption inhibitors was also observed in BRAF inhibitor-resistant melanoma cells, which might be explained by the up-regulation of Cyclin-B1 expression in resistant cells [193]. The above findings also implicate that tumor-acquired resistance to certain pathway inhibitors may correlate to the abnormally expressed KIFs, given the fact that over-expression of KIFs mediates resistance to anti-mitotic drugs like docetaxel and paclitaxel [194–197], whether these aberrant KIFs can serve as biomarkers to predict vulnerability to pathway inhibitors remains unclear. Since many KIFs reviewed in this paper are closely associated with the activation of Akt signaling axis, which was mainly represented by the elevation of phosphorylation levels of Akt pathway proteins. And it was reported that there was a close relationship between the phosphorylation levels of Akt pathway proteins and the responsiveness to pathway inhibitors [189]. Therefore, further clarification of the intrinsic mechanism of abnormally expressed KIFs-induced Akt activation might be helpful to guide Akt inhibitor therapy, such as the mechanism of KIFs-mediated up-regulation of Akt pathway phosphoproteins. Additionally, digging into the genetic alterations of KIFs may also contribute to the development of biomarkers to predict susceptibility to certain targeted therapeutic agents, since tumors carrying rearranged during transfection (RET) oncogene fusions display a higher sensitivity to RET tyrosine kinase inhibitors, especially the most frequent and well characterized KIF5B-RET fusion variant in non-small cell lung cancer [198, 199]. Hence, whether other KIFs-associated genetic variations can also affect the reactivity to pathway inhibitors deserves further investigation.

In addition to the feasibility of KIFs-targeting combined with pathway inhibition in cancer therapy, as well as the potential influence of aberrant KIFs on drug sensitivity, the abnormally expressed KIFs are also demonstrated to be utilized for early cancer diagnosis and to instruct preventive treatment. For example, the KIFC1-involved three genes expression score could predict the brain metastasis risk for NSCLC patients and identify the high-risk individuals who might benefit from prophylactic intervention of the central nervous system [200]. By mimicking the interactions between different cell groups in the tumor microenvironment of pancreatic cancer, with the help of high-throughput mass spectrometry analysis of extracellular vesicle (EV), the kinesin-1 family member KIF5B was determined as a genuine early biomarker for advanced pancreatic cancer [201].

To sum up, in-depth understanding of disordered KIFs in tumor progression is of great benefit to cancer diagnosis, prognosis evaluation, as well as precision treatment. However, relevant exploration and cognition are still insufficient for HBCs. Further clarifying the specific roles of KIFs in tumor progression and developing targeted therapeutic drugs have become the focus of subsequent research on HBCs. Although many challenges lie ahead, such as tumor heterogeneity, drug resistance, reasonable drug compatibility, and effective clinical translation, it’s just like the darkness before the dawn, there are already forward lights, like the success of Arry-520 for multiple myeloma treatment. In the future, with the deepening acquaintance of the intermolecular regulatory network among kinesin family members, as well as the exploration and development of novel KIF targets, there may be corresponding answers to the above questions. Anyway, targeting of KIFs for anti-mitotic therapy is worth trying, although the road may be difficult and tortuous.

### Electronic supplementary material

Below is the link to the electronic supplementary material.

**Supplementary Figure 1**. Several major KIFs-associated signaling pathways. Akt signaling: KIFC3, KIF3B, KIF14, KIF4A, KIF18A, KIF26B, and KIFC1 in HCC; KIF11 and KIF15 in GBC. WNT/β-catenin signaling: KIF11, KIF23, KIF18B, KIF2C, and KIFC1 in HCC. MEK/ERK signaling: KIF15 and KIF2C in HCC.

## Data Availability

Not applicable.

## Glossary

HBC: Hepatobiliary carcinoma
HCC: Hepatocellular carcinoma
CCA: Cholangiocarcinoma
GBC: Gallbladder carcinoma
ATPase: Adenosine triphosphatase
ALS: Amyotrophic lateral sclerosis
HSP: Hereditary spastic paraplegia
QF-DE: Quick-freezing and deep-etching
siRNA: Small interfering RNA
SNP: Single nucleotide polymorphism
HBV: Hepatitis B virus
co-IP: Co-immunoprecipitation
ASPM: Abnormal spindle-like microcephaly-associated protein
Hh: Hedgehog
H3K27ac: Acetylation of histone H3 lysine 27
PSMD12: Proteasome 26 S subunit, non-ATPase 12
PHGDH: Phosphoglycerate dehydrogenase
ROS: Reactive oxygen species
TBC1D7: Tre2-Bub2-Cdc16 (TBC) 1 domain family member 7
LC-MS: Liquid chromatograph mass spectrometry
TSC: Tuberous sclerosis complex
EMT: Epithelial-mesenchymal transition
HMGA1: High Mobility Group AT-Hook 1
BTC: Biliary tract carcinoma
IHC: Immunohistochemistry
OS: Overall survival
RFS: Relapse-free survival
DFS: Disease-free survival
TFS: Tumor-free survival
Kaplan-Meier: K-M
PS: Performance status
PVTT: Portal vein tumor thrombus
TMA: Tissue microarrays
TCGA: The Cancer Genome Atlas
GEO: Gene Expression Omnibus
ICGC: International Cancer Genome Consortium
OV: Opisthorchis viverrini
NDMA: N-nitrosodimethylamine
MTAs: Microtubule-targeted agents
NSCLC: Non-small cell lung cancer
AR: Androgen receptor
KSP: Kinesin spindle protein
STLC: S-Trityl-L-cysteine
AS-2: Adociasulfate-2
RNAi: RNA interference
CDX: Cell line-derived xenograft
PDX: Patient-derived xenograft
SQAG: Sulfoquinovosylacylglycerol
TNBC: Triple negative breast cancer
TKI: Tyrosine kinase inhibitor
SAC: Spindle assembly checkpoint
RET: Rearranged during transfection
EV: Extracellular vesicle
