# Supplementary material for: The role of kinesin family members in hepatobiliary carcinomas: from bench to bedside
Source: Biomark Res. 2024 Mar 3;12:30. doi: 10.1186/s40364-024-00559-z (PMC10910842; doi:10.1186/s40364-024-00559-z)
Supplement: Supplementary file 1 — Supplementary Figure 1. Several major KIFs-associated signaling pathways. Akt signaling: KIFC3, KIF3B, KIF14, KIF4A, KIF18A, KIF26B, and KIFC1 in HCC; KIF11 and KIF15 in GBC. WNT/β-catenin signaling: KIF11, KIF23, KIF18B, KIF2C, and KIFC1 in HCC. MEK/ERK signaling: KIF15 and KIF2C in HCC. [file 40364_2024_559_MOESM1_ESM.docx]

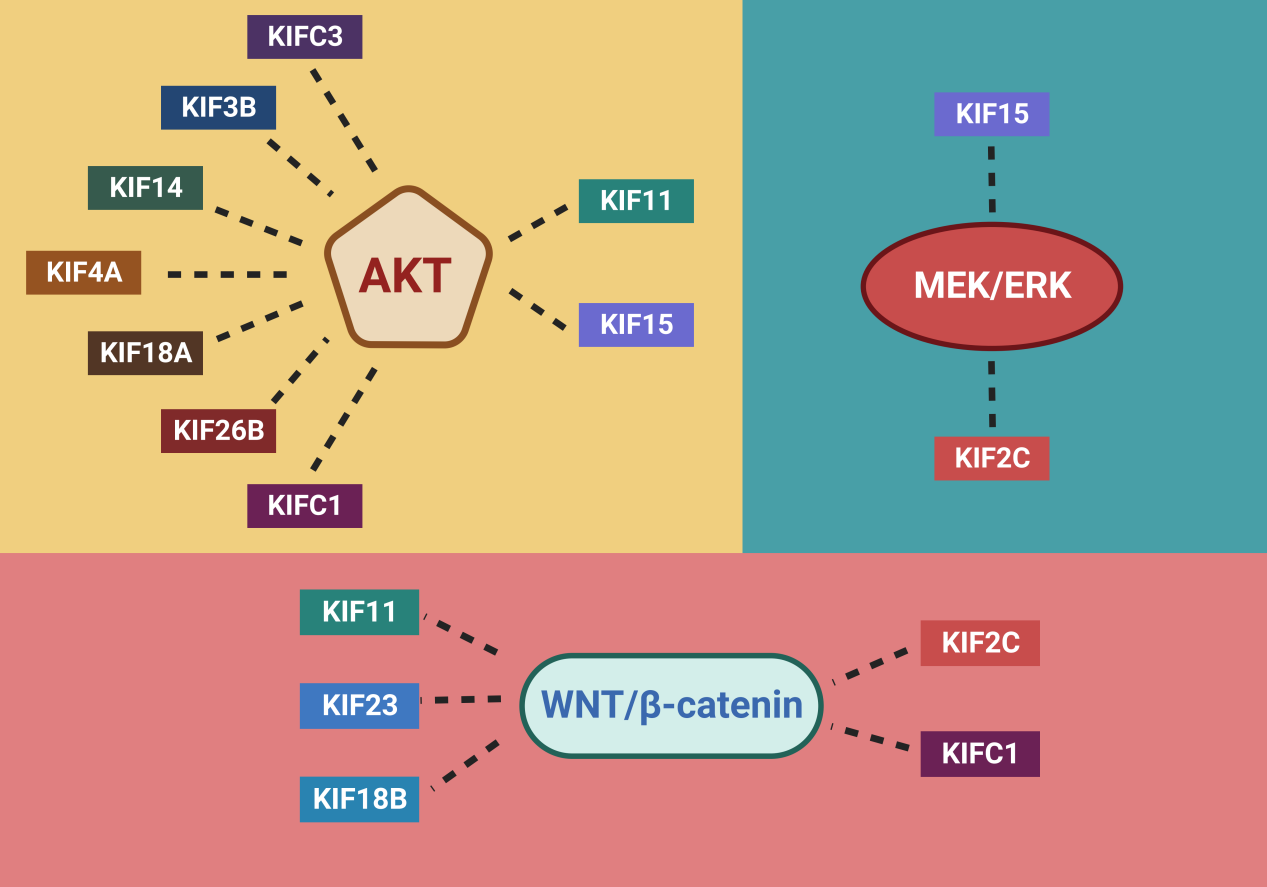


**Supplementary Figure 1. Several major KIFs-associated signaling pathways.**

Akt signaling: KIFC3, KIF3B, KIF14, KIF4A, KIF18A, KIF26B, and KIFC1 in HCC; KIF11 and KIF15 in GBC.

WNT/β-catenin signaling: KIF11, KIF23, KIF18B, KIF2C, and KIFC1 in HCC.

MEK/ERK signaling: KIF15 and KIF2C in HCC.
